# Towards Transparency by Design for Artificial Intelligence

**DOI:** 10.1007/s11948-020-00276-4

**Published:** 2020-11-16

**Authors:** Heike Felzmann, Eduard Fosch-Villaronga, Christoph Lutz, Aurelia Tamò-Larrieux

**Affiliations:** 1 Centre of Bioethical Research and Analysis (COBRA), NUI Galway, Galway, Ireland; 2 eLaw Center for Law and Digital Technologies, Leiden University, Leiden, The Netherlands; 3 Nordic Centre for Internet and Society (NCIS), BI Norwegian Business School, Oslo, Norway; 4 Forschungsinstitut für Arbeit und Arbeitswelten (FAA-HSG), University of St. Gallen, St. Gallen, Switzerland

**Keywords:** Transparency, Artificial intelligence, Framework, Automated decision-making, Accountability, Design, Interdisciplinary, Ethics

## Abstract

In this article, we develop the concept of Transparency by Design that serves as practical guidance in helping promote the beneficial functions of transparency while mitigating its challenges in automated-decision making (ADM) environments. With the rise of artificial intelligence (AI) and the ability of AI systems to make automated and self-learned decisions, a call for transparency of how such systems reach decisions has echoed within academic and policy circles. The term transparency, however, relates to multiple concepts, fulfills many functions, and holds different promises that struggle to be realized in concrete applications. Indeed, the complexity of transparency for ADM shows tension between transparency as a normative ideal and its translation to practical application. To address this tension, we first conduct a review of transparency, analyzing its challenges and limitations concerning automated decision-making practices. We then look at the lessons learned from the development of Privacy by Design, as a basis for developing the Transparency by Design principles. Finally, we propose a set of nine principles to cover relevant contextual, technical, informational, and stakeholder-sensitive considerations. Transparency by Design is a model that helps organizations design transparent AI systems, by integrating these principles in a step-by-step manner and as an ex-ante value, not as an afterthought.

## Introduction

The rise of machine learning and artificial intelligence (AI) has led to the creation of systems that can reach largely autonomous decisions, such as AI-based diagnostic tools for health applications (e.g., detection of diabetic retinopathy, cf. Abràmoff et al. 2018), recommender systems (e.g., YouTube recommender algorithms, cf. Bishop 2018), or predictive policing and criminal sentencing (Brayne 2017; Brayne and Christin 2020). While traditionally algorithms had to be programmed ‘by hand’ with rules to follow and weights to attach to specific data points, machine learning algorithms have changed the way patterns are extracted from datasets and how predictions are made (Van Otterlo 2013).

In this article, we are interested in the delegation of a decision-making process to an algorithm, i.e., automated decision-making (AlgorithmWatch 2019). We understand automated decision-making (ADM) as a subpart of AI, an automated process with no human involvement to reach a decision (Karanasiou and Pinotsis 2017; ICO 2020). We start with the premise that automated decision-making algorithms “make generally reliable (but subjective and not necessarily correct) decisions based upon complex rules that challenge or confound human capacities for action and comprehension” (Mittelstadt et al. 2016, p. 3). Automated decision making-systems can have impacts on individuals and society at large, creating novel ethical challenges that open up fundamental questions regarding responsibility, human dignity, and the relation between humans and machines (Coeckelbergh 2020; Matthias 2004; Latonero 2018). From a legal perspective, automated decision-making systems’ potential adverse impact on individuals and society necessitates regulatory reaction. It is thus not unsurprising that automated decision-making systems that produce legal effects (e.g., criminal sentences) or otherwise significantly impact an individual (e.g., being denied a loan) are—depending on whose legal opinion one follows—either forbidden in European data protection law or at the minimum the individual has a right not to be subjected to it. Numerous (legal) scholars have debated how transparent such systems must be respectively whether a right to having access to the logic involved in the decision-making process should exist (Casey et al. 2019; Edwards and Veale 2018; Felzmann et al. 2019a, b; Goodman and Flaxman 2017; Kaminski 2019; Selbst and Powels 2017; Wachter and Mittelstadt 2019; Lepri et al. 2018). The literature indicates that the term transparency relates to multiple concepts, fulfills many functions, and holds different promises and that transparency is becoming an important aspect of the regulatory discourse on AI (European Commission 2020).

The complexity of transparency in automated decision-making systems shows tension between transparency as a normative ideal and its translation to practical application (Felzmann et al. 2019a). As literature on algorithmic culture has highlighted, algorithms should not be seen as clearly delimited, neutral constituents of information technologies, or “conceptual objects indifferent to implementation details”, but are “heterogeneous and diffuse sociotechnical systems” (Seaver 2017, p. 1), instantiated and imbued with meaning through their embeddedness in those technologies, and with their various and often fluid and evolving roles merging technical and cultural practices (Dourish 2016; Roberge and Seyfert 2016; Seaver 2017). The concept of “data assemblages” (Kitchin and Lauriault 2014) captures this contextuality and fluidity of data-based technologies, including algorithms (see Siles et al. 2020 for a recent application of the concept to the Spotify recommendation algorithm). It calls for the study of such systems from a critical and holistic perspective, including the consideration of the political economy in which data assemblages enfold, systems of thought, forms of knowledge, governmental and legal aspects, practices, places as well as subjectivities and communities. As Ted Striphas (2015) points out, with reference to the impact of Amazon’s recommendation system on the visibility of LGBT literature, the dimension of culture also needs to be recognised in analyses of algorithms, insofar as cultural work is increasingly being offloaded onto frequently privatised algorithms and information technologies, under the guise of neutral technological decision-making. These are often intransparent not just to outsiders, who may be deliberately excluded from knowledge by private access barriers, but also to insiders, with no-one clearly holding comprehensive explanatory knowledge about their functioning (Seaver 2019). These insights need to inform discussions of transparency, which have to account not just for transparency of the technical features of an algorithm itself, but also for its practical implementation within existing social structures and its assigned cultural meanings. Increasing the complexity of the analysis further, Nick Seaver (2017) highlights that the very attempt at making algorithms transparent and accountable will change the social scene within which they operate.

Therefore, practical guidance on design for transparency that is sensitive to the wider roles and social embeddedness of ADM is necessary to help promote the beneficial functions of transparency while mitigating its challenges. Building upon the idea of “Transparency by Design” (TbD) as an emerging concept (Hildebrandt 2013; Mascharka et al. 2018), we aim to provide such a roadmap addressed especially towards those tasked with the development of ADM systems.

We start this article by describing the functions and challenges of transparency in relation to automated decision-making practices (Sect. 2). In Sect. 3, we describe the TbD model and elaborate on its key functions. We have selected nine principles to cover relevant contextual, technical, informational, and stakeholder-sensitive considerations. The TbD model integrates these principles in a step-by-step manner. Our model places transparency not as an afterthought but as an ex-ante value to be taken into consideration from the very beginning when designing AI systems. We intend the TbD principles as guidance for organizations and AI developers who are increasingly faced with demands to realize transparency for their AI systems.

## Transparency: A Multi-faceted Term

### Defining Transparency: Integrating Three Perspectives

Transparency is a complex construct that evades simple definitions. It can refer to explainability, interpretability, openness, accessibility, and visibility, among others (Felzmann et al. 2019b; Weller 2017). Moreover, different disciplines stress different aspects and virtues of transparency.^1^ “Economists regard transparency as a precondition for optimal markets, political scientists conceptualize it as a precondition for political participation, and legal scholars stress that transparency is a precondition for administrative legality” (Meijer 2014, p. 510). While all the disciplines seem to agree that transparency adds a positive value, it is often not clear what it means, to whom it relates, and to what extent it is beneficial.

Our discussion is based on the assumption that the purely informational perspective on transparency, while an essential component of any understanding of transparency, is falling substantially short, insofar as it ignores the deeply value-laden embeddedness of transparency into individual agency, relational and systemic practices. The significance of transparency cannot be understood when it is merely conceived as the transfer of information from one agent to another, without attention to the meanings, values and social functions associated with such transfer. We differentiate with Albert Meijer (2014) between three broad perspectives on transparency which foreground the underpinning normative and social character of transparency practices: Transparency as a virtue, a relation, and a system. The first understanding of transparency as a virtue is a normative notion, identifying standards for evaluating public actors’ behavior (Meijer 2014). Transparency is seen as an intrinsically valuable characteristic of agents, systems, or organizations and consists of the consistent openness about their operations, behavior, intentions, or considerations. However, the notion of transparency as a virtue does not specify the target or audience to whom an actor is transparent. Here, the relational notion of transparency comes in. Within this relational perspective, transparency is conceived not as an individual characteristic but as a relation between an agent and a recipient. Transparency cannot be understood outside this relation. Accordingly, it is not sufficient if agents show openness about their operations; how their openness is received and understood by the recipient is equally important. This relational understanding of transparency is reflected in the definition of transparency “as the availability of information about an actor allowing other actors to monitor the workings or performance of this actor” (Meijer 2014, p. 511). Finally, the systemic perspective takes into account the institutional context of the relations of transparency. Awareness of the institutional context and the embeddedness of transparency communications within the specific characteristics of this context, including associated legal, regulatory and organizational measures, is essential for a realistic understanding of its likely practical impact and its effective implementation.

Throughout this paper, we consider the integration of all three perspectives to be essential for a full understanding of transparency and the role it plays in ADM environments. Understanding transparency as a normative ideal or virtue recognizes the importance of embedding it deeply in the DNA of a system and an organization. The primary concern in the relational understanding of transparency is the need to ensure that transparency measures are always designed and assessed with regard to their impact on stakeholders. Finally, transparency according to the systemic perspective considers the institutional embeddedness in the implementation context. This includes especially the role and effectiveness of accountability measures.

### Positive Aspects and Outcomes of Transparency

There is a widespread perception that transparency is beneficial to allocate resources efficiently and to render information holders more accountable (Forssbaeck and Oxelheim 2014). In much of the literature on transparency, the emphasis of transparency as a positive force relies on the informational perspective, which connects transparency to the disclosure of information (Tielenburg 2018) instead of connecting it to a broader and multi-dimensional perspective that considers informational, virtue-based, relational, and systemic aspects together (Felzmann et al. 2019b). Within the informational perspective, transparency is typically seen as a means to overcome information asymmetries (Carlsson 2014; Forssbaeck and Oxelheim 2014). Transferring information from the private to the public sphere (i.e., making the information open and accessible) reduces information asymmetries. A state of transparency does not imply complete information but merely refers to the state in which there is no *problematic* information asymmetry. In such a state, “no one has the advantage of being better (privately) informed” (Forssbaeck and Oxelheim 2014, p. 6). Public disclosure may thus serve as an equalizing function since it does not merely ensure information sharing between two parties via an exercise of bargaining power but guarantees everyone has access to the relevant information regarding the inner workings of a process or an organization (Berglund 2014). In order to achieve this state of transparency, “transparency will thus require full disclosure of all relevant information in a timely manner” (Berglund 2014, p. 360; see also Rawlins 2008; Williams 2005). The informational approach to transparency aligns with the principal-agent theory, where the principal, who delegated certain tasks to an agent, needs to obtain information in order to check if the agent holds up their part of the contract (Eisenhardt 1989). By doing so, the principal can reduce the likelihood of problems such as adverse selection or moral hazard (Berglund 2014; Forssbaeck and Oxelheim 2014; Tielenburg 2018). According to the informational and principal-agent perspective, transparency connects to several positive consequences, such as fostering trust, facilitating accountability, supporting autonomy, and allowing a greater level of control, which we discuss in more detail in the following paragraphs.

The informational approach to transparency serves as a precondition to enable other desirable functions necessary in an environment surrounded by ADM systems. First, transparency-as-information in AI connects strongly to explanations and explainability (Veale et al. 2018). Someone involved in a decision-making process should be able to have access ex-ante to information about the quality or intent of a process and ex-post about the outcomes and how they came about (Felzmann et al. 2019a) The topic of explainable AI (XAI) has gained much attention in recent years and has become an active field of inquiry (Abdul et al. 2018; Adadi and Berrada 2018; Edwards and Veale 2017, 2018; Miller 2019; Pasquale 2015; Zerilli et al. 2019). Legal scholars have debated whether and what kind of explanation the General Data Protection Regulation’s (GDPR) right not to be subject to certain kinds of fully automated decisions (Art. 22) requires, in light of the right of individuals to obtain “meaningful information about the logic involved, as well as the significance and the envisaged consequences” (Art. 15(1)(h) GDPR) of the automated processing occurring (Casey et al. 2019; Edwards and Veale 2017, 2018; Goodman and Flaxman 2017; Kaminski 2019; Selbst and Powles 2017; Wachter et al. 2017). In professional standards and best practices, the informational perspective of transparency also translates into the concept of “inspectability” (Zerilli et al. 2019). Inspectability can be understood as allowing a third party to examine a system to ensure they meet defined standards of decision-making. Using other, closely related labels, such as verifiability or traceability, several relevant organizations refer to it as one of the core aspects of transparency. The IEEE’s report on ethically aligned design for autonomous and intelligent systems (A/IS) (IEEE 2019), for example, features transparency as one of eight overarching principles, and so did the EC White paper on artificial intelligence (2020). The report specifies that transparency is the ability to discover the basis of a decision made by an A/IS, and relates transparency to traceability, verifiability, intelligibility, and honest design (IEEE 2019).

Second, the informational perspective also links transparency to accountability. Already in 1913, Supreme Court Justice Louis Brandeis stated that “sunlight is the best disinfectant” (Fox 2007, p. 664). The connection between transparency and accountability refers to the idea that any observation into a system’s logic provides insight, and this insight creates knowledge, which in turn is a precondition for holding systems accountable (Ananny and Crawford 2018; Tielenburg 2018; Zarsky 2013). However, this statement has some drawbacks, as it does not hold true all times (Kolkman 2020). Thus, transparency and accountability are strongly connected but not synonymous (IEEE 2019; Meijer 2014; Zarsky 2013). Accountability refers to “a relationship between an actor and a forum, in which the actor has an obligation to explain and to justify his or her conduct, the forum can pose questions and pass judgment, and the actor may face the consequences” (Bovens 2007, p. 447). This definition highlights the various interacting elements of accountability: the actor, the forum, the relationship between the actor and forum, the content and criteria of the account, and finally, the consequences that can be imposed (Wieringa 2020), which connects it necessarily to a broader multidimensional understanding of the concept. The forum—or whom transparency is directed to—will necessarily shape the form and content of how to ensure accountability. Accounts can only be given if the audience understands the subject matter and can engage with information provided in a critical way (Kemper and Kolkman 2019). However, accountability is also broader than transparency, as transparency merely refers to the “transparent workings of a system” and does not say “why this system was deemed ‘good enough’ at decision making” (Wieringa 2020, p. 4). For the relationship among actors and the forum, transparency is key concerning the information-giving process, which in turn enables the discussion and deliberation on what consequences should be imposed in a given situation. Concerning ADM often proposed ex-ante approaches (e.g., impact assessments) or ex-post ones (e.g., analysis of the impact of a final decision) have been criticized to as not modular enough and not taking the entire process (design, implementation, evaluation) into account (Wieringa with reference to Neyland 2016; Diakopoulos 2016; Kroll et al. 2016).

Third, another frequently discussed outcome or benefit of transparency is trust (Elia 2009). We understand trust as “a psychological state comprising the intention to accept vulnerability based upon positive expectations of the intentions or behavior of another” (Rousseau et al. 1998, p. 395). Trust involves a trustor’s/principal’s assessment of the trustee/agent’s trustworthiness in terms of ability, benevolence, and integrity (Bhattacherjee 2001; Jones 1996). Transparency can signal not only the ability to perform as expected or promised but also seems connected to integrity, insofar as actors that are willing to disclose information about themselves transparently may be thereby seen to convey their integrity. Empirical studies have investigated the transparency-trust nexus; Elia (2009) summarizes this literature in a business context, pointing to positive effects of transparency such as social capital gains, increased cooperative behavior, and reputation spillover effects. However, the relationship between transparency and trust remains somewhat contested and ambivalent, also in the context of technologies such as AI and social robots (Felzmann et al. 2019a, b).

### Challenges and Limitations of Transparency

Despite these positive aspects and functions of transparency, an increasing body of literature has pointed to issues associated with the concept (Ananny and Crawford 2018; De Laat 2018; Tielenburg 2018). A vital criticism of an informational view of transparency is that it often neglects the demand-side of transparency (Forssbaeck and Oxelheim 2014). The information does not only need to be disclosed, but it also needs to be received, interpreted and understood by the respective audience (Kemper and Kolkman 2019). For information disclosure to lead to transparency, the information must be adapted to the audience’s needs (Tielenburg 2018).

The informational perspective sees transparency as mostly static: the information is already present and available to those asked to disclose it. A transparency requirement will only ensure that this same information will then be disclosed to others (Ananny and Crawford 2018, pp. 974–975). The focus rests on the availability of information and not so much on reflection on the nature and context of this information and the process of how information is obtained and made available (Tielenburg 2018). Information, in this understanding, is seen as neutral facts instead of socially constructed and interpreted artifacts (Tsoukas 1997). Little consideration is given to the selection mechanisms, processes, and politics behind the disclosure (Albu and Flyverbom 2019). However, “[e]fforts to provide transparency are fundamentally performative […] they do not create neutral knowledge about and observations of organizations, but rearrange them in unexpected ways” (Ringel 2019, p. 706).

Similar criticism is found in the literature about the explainability of AI. Despite the promising research in XAI and FAccT,^2^ substantial challenges to making AI more transparent remain. Ananny and Crawford (2018), as well as de Laat (2018), provide an excellent overview of such challenges. One key challenge and obstacle of transparency in AI is the complexity of the underlying technology. Modern AI systems often rely on machine learning such as neural networks and support vector machines. Especially with more advanced configurations and large amounts of training data, such systems become virtually impossible to trace step-by-step (Burrell 2016), even for experts. Thus, a trade-off must be made between accuracy and explainability or interpretability (Adadi and Berrada 2018), as advanced systems that are more accurate in their predictions are becoming less interpretable. Drawing on Hirschman’s theory of voice and exit (1970), it can be argued that transparent information disclosure by organizations about their ADM systems increases consumers’ ability to make decisions, but depending on the practical implementation of transparency and the information gained, consumers may become either more motivated to exit (stop using the system) or use voice (keep engaged, but push for a change). However, given the normative, relational and social dimensions of transparency, the fact that the organisation offers transparency may in itself contribute to increased relational trust and loyalty, thereby decreasing the wish to exit, even though potentially these systems might serve users less effectively than less transparent but more accurate systems.

Another issue relates to privacy. Making AI systems fully transparent can expose sensitive and private data, particularly if the underlying training data is published (Ananny and Crawford 2018; De Laat 2018). This is a problem especially if personal data is used for training machine learning algorithms, such as voice recordings, emails, social media posts, and pictures. Given that for less biased algorithms more diverse and representative training data sets are necessary, which includes data from vulnerable population groups, publishing such data could expose those who are already disadvantaged to harm. Hence, calls for transparency and openness have to contend with individuals’ right for privacy. Moreover, transparency can be disadvantageous for companies from a competition perspective. Companies can argue that making their AI systems more transparent could result in competitors copying these systems. It could also allow users and competitors—or nefarious entities – to better target interventions to game or sabotage the systems.

As shown above, increased trust by users and consumers has been a critical argument for transparency projects among proponents working from an informational perspective (Hood 2006). While this association between transparency and trust makes intuitive sense, the empirical facts are less clear (Meijer 2014). Transparency can foster trust but does not necessarily have to (Albu and Flyverbom 2019; Greiling 2014). Empirical research on the transparency-trust nexus in general has shown mixed results. For example, in the context of government transparency, initiatives such as Freedom of Information requirements have frequently not increased citizen trust, and in some cases even led to declines in trust (Grimmelikhuijsen et al. 2013, Foster and Frieden 2017). Those who question the positive influence of transparency on trust stress that transparency can lead to confusion and uncertainty when the information provided is not easily understandable to the audience, and that other factors outside of transparency may be more important for trust (O’Neill 2002). In a series of experiments across two cultures (South Korea, Netherlands), Stephan Grimmelikhuijsen and colleagues (2013) found mixed and partly adverse effects of transparency on different dimensions of trust. This shows that transparency is just one factor among several that might affect trust, and that the importance of individual meanings, specific situational factors and cultural context may override transparency or even invert its significance from positive to negative. In the more specific context of AI systems, an overview of empirical literature on the effects of transparency on other constructs, including trust, is available in Heike Felzmann and colleagues (2019a). The overview suggests that the trust-generating capacity of transparency in AI systems depends on different factors, such as user expectations, how transparency is implemented (Rader et al. 2018) and which technologies are investigated (Kulesza et al. 2013). Work by Eslami and colleagues (2018) on transparency and online behavioral advertising shows how transparency can lead to algorithmic disillusionment and disappointment, rather than trust.

The positive association between transparency and accountability can also be called into question (Meijer 2014). This is particularly true in situations where the agency is distributed and complex (Nissenbaum 1996), such as in the case of Wikipedia, where dozens of individuals can contribute to an article and bots are increasingly involved in the editing process. Even though the edits are transparently documented in the history and discussion section, the accountability mechanisms for wrong, irrelevant, and misleading information are far from clear. The person or bot posting the wrong, irrelevant, or misleading information might not have to justify their behavior and might not face sanctions. Thus, whether transparency translates into accountability depends on the institutional context, the power structures, the demand and push for accountability and other factors (Ananny and Crawford 2018). A system can be transparent, in the sense that it is mostly devoid of information asymmetries, but its creators may still avoid accountability due to their position of power or lack of awareness and action by its users. “If transparency has no meaningful effects, then the idea of transparency can lose its purpose. […] Visibility carries risks for the goal of accountability if there is no system ready and ‘capable of processing, digesting, and using the information’ to create change” (Ananny and Crawford 2018, p. 978; referring to Heald 2006).

Finally, transparency, autonomy, and control might be less clearly linked than a purely functional perspective might assume. If the audience cannot leverage the information because it remains difficult to access, is presented in a complex and obscure way, or they have no meaningful alternative to the service, no increase in autonomy and control is obtained. As the literature on privacy policies and informed consent has shown, transparency can be used as a means for user-responsibilization, shifting the responsibility increasingly to ill-equipped and overwhelmed users and consumers, who in practice end up mostly ignoring information that remains meaningless for them (Ben-Shahar and Schneider 2011, 2014; Calo 2011). It has been argued that new modes of delivering information are required to facilitate the intended increase in autonomy and control through transparency (Calo 2011).

### Example of Transparency and Automated-Decision Making Systems

To illustrate the points made in the previous sections, we discuss a current example of ADM systems and how transparency tensions apply. This is by no means a comprehensive overview but an attempt to show transparency in action in the context of automated decision-making systems more clearly.

An example of transparency struggles when it comes to ADM is the domain of content moderation on social media (Gillespie 2018; Roberts 2019). Social media platforms such as Facebook and Twitter use a mix of AI-based automated decision-making and human labor to flag and remove content that violates the platform policies (Singh 2019). However, how platforms make such decisions, and the role of automated versus human intervention remains mostly opaque. Users have shown confusion and frustration about the platforms’ content moderation decisions (Suzor et al. 2019) and calls for greater transparency have been made (Leetaru 2018). Transparency in this context can be on an individual level or a systemic level. On an individual level, transparency means that users who have been affected by a content moderation decision receive sufficient and contextualized explanations of how and why a decision was made (Suzor et al. 2019). On a systemic level, transparency means aggregated insights on how many decisions are made, based on which violations these decisions were made, and who and how decisions are made in general.

Due to public pressure (e.g., Santa Clara Principles 2018), some social media platforms now release transparency reports to address the system transparency demands but such accounts have been criticized for being mostly performative, while individuals are often left in the dark how content moderation came about and how a decision can be followed up on (Suzor et al. 2019). This means that even from an informational perspective alone, the transparency provided is insufficient. In addition, if looking at transparency through the lens of the categories of virtue, relational meaning and systemic embeddedness, further challenges arise. While transparency could be seen as an expression of a virtue, this interpretation depends on the assumption that the meaning of transparency practices is of an ethical nature. While social media companies frequently accompany transparency actions with expressions of moral responsibility, not only do these avowed ethical motives not necessarily match their wider patterns of action, but they also appear incoherently implemented. From a relational perspective, it would matter in particular how transparency measures affect the relationship between users and the company. It appears that users are often dissatisfied with the type of transparency report available to them, but at the same time feel helpless with regard to where to take their discontent. Not only is it difficult to obtain meaningful responses to queries or concerns regarding content management decisions, but more generally the individual user is given little reason to think that they have any means to influence social media companies’ practices in this field. Suzor and colleagues (2019) discuss several measures of how transparency could be enhanced from a relational perspective, such as “more detailed and individualized explanations of the content moderation process” (p. 1537). This includes explanations of why a particular user is being flagged and based on which exact post or passage, but it could also include more general information on the implementation of content moderation at the interface between algorithmic and human decision-making, including the publication of training material that human content moderators use and an overview of the training data. Moreover, platforms should “help users understand how rules are created and by whom; how moderators learn and enforce the rules; what the composition, training, and working conditions of moderation teams are like; how the platform ensures consistency; how consistent decisions are in practice; and how mistakes and novel issues are dealt with” (p. 1536). Such more comprehensive transparency might address the relational challenges arising from potentially contentious content moderation decisions. And finally, the systemic perspective would draw attention to the wider embeddedness of transparency practices within the social media company and how they are implemented in the societal context. Rather than merely focusing on the information provided, transparency should be integrated with more far-reaching accountability measures, addressing aspects such as bias and undue influence. Such a wider understanding of transparency would involve “large-scale access to data on individual moderation decisions as well as deep qualitative analyses of the automated and human processes that platforms deploy internally” (p. 1538), but also consider their effectiveness in achieving accountability aims. In terms of the actual infrastructure, technology, and human labor that go into content moderation, social media platforms are also notoriously intransparent and, while appearing to provide a disembodied digital service, they actually outsource much of the human labor to countries like the Philippines, India, and Mexico (Roberts 2019), frequently with problematic labour practices vis-a-vis the often psychological burdensome or even traumatic nature of the required work.

## Transparency by Design

### A Model for Transparency by Design

The term Transparency by Design (TbD) is inspired by the Privacy by Design (PbD) framework. Cavoukian (2009) coined the term PbD and envisaged a holistic and integrative take on privacy protection to help companies consider the benefits of privacy and counter the assumption that privacy protection ultimately can only be implemented at the expense of other values. While Cavoukian’s principles were established in the 1990s, they have recently regained momentum, as PbD was codified within the GDPR as data protection by design (Art. 25 GDPR). Thereby, system developers established in the European Union or targeting individuals in the Union must ensure that all the principles of data protection law are ensured through organizational and technical measures during the design process of their systems. Ways to fulfill the requirement of the GDPR have been proposed (Tamò-Larrieux 2018).

Already before its codification within the GDPR, scholars further elaborated privacy engineering guidelines (Cavoukian et al. 2014; Dennedy et al. 2014) that concretize PbD principles or have re-envisioned PbD as value-sensitive design (Mulligan and King 2011). While PbD is probably the most widely known approach to integrating value considerations into the design process, wider consideration of values beyond privacy alone has been proposed by various authors, including most prominently Friedman et al.’s (2008) Value-Sensitive Design (VSD) methodology, Phillip Brey’s (2010) disclosive computer ethics, or more recently the European Commission’s guidelines on trustworthy AI (HLEG AI 2019). There have also been various calls for a broadly value-encompassing Ethics by Design approach, a term that appears to become increasingly popular (d’Aquin et al. 2018; Dignum et al. 2018; Iphofen and Kritikos 2019). However, so far there is no distinct, unified methodology associated with this term and these proposals seem functionally equivalent to other more broadly value oriented approaches.

We understand TbD as combining a specific and ultimately potentially codifiable approach, similar to PbD, with considerations that address the significance of wider normative, relational and social factors in order to achieve a meaningful realisation of transparency. As outlined above, achieving transparency requires attention to individual perceptions, the positioning of actors vis-a-vis each other, and their embeddedness in wider social structures that are essential to give meaning to transparency practices. Nevertheless, we consider PbD a particularly valuable model as contextual framework for TbD, based on three key takeaways from the discussion around PbD (see below outlined TbD-framework): first, PbD started with broad principles that have evolved into more concrete principles codified in the law. This shows that the realization of an ideal can find a way into law, thereby becoming binding if proven useful. By focusing on the development of guidance that outlines concrete steps for the realisation of different principles underpinning TbD we aim to provide a level of practical usability that might allow a similar evolution towards eventual codification. Second, the design of technologies significantly impacts the subsequent use of these technologies and their effects on people (Kudina and Verbeek 2019). Thus, governing the design process to address such effects needs to be an important goal for those who want to ensure the responsible implementation of information technologies in society and the protection of citizens from unintended negative consequences. TbD aims to do justice to the challenges for effective governance arising from the technical complexity and societal significance of transparency for AI systems by including attention to the continuum from design to implementation in the framework. Third, PbD has an inherent balancing nature linked to it: When implementing privacy through technical and organizational measures efforts must be made to integrate privacy carefully with other values from the outset so that privacy realisation is not just perceived as a post hoc limiting factor on other values but as a core value that is balanced against and realised together with other values throughout the design process. PbD is premised on the assumption that systems resulting from such a careful balancing approach to design will integrate privacy not with a zero-sum, but positive-sum result. Similarly, for TbD, we believe that the existing challenges to achieving transparency should not be seen to simply make a TbD approach more onerous, but rather to allow a more careful appreciation of what implementations of transparency may offer the most overall value for users or consumers.

### Transparency by Design: Phases and Principles

Merely calling for transparency of AI systems is ineffective in practice (Zarsky 2013). The requirements of transparency must be translated into practical steps. The aim of the TbD principles is to provide such practical guidance. Our model is inspired by PbD and Tal Z. Zarsky’s (2013) taxonomy for transparency of predictive analytics, which focuses on the information flow, and ultimately divides the transparency processes into three segments: (1) design of AI systems: general design requirements to enhance transparency when developing new systems, (2) information on Data Processing and Analysis: information provision that makes data processing, decision-making routines and risks more transparent once the system is in use, and (3) accountability: the organizational and stakeholder-oriented transparency aspects in terms of inspectability, responsiveness and reporting routines. Thus, our model focuses on general design requirements, user-oriented information provision about the system, and the management of transparency for systems in an organizational sense.

**Fig. 1:**
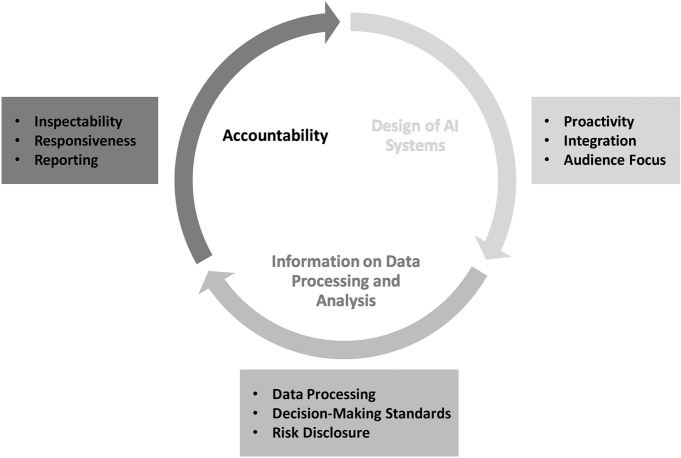
A Model for transparency by design

While the TbD-framework rests on those three phases, we derive the content of each principle within these phases from the academic literature on transparency as well as policy and industry reports on transparency in AI systems. We propose nine principles, aligned with the three phases outlined in Fig. 1.

Ultimately, the goal of the TbD-framework is to promote the beneficial aspects of transparency and simultaneously mitigate its challenges. These principles frame the obligations that system designers, especially engineers, and organizational stakeholders face when integrating the transparency requirement in the development of AI systems (Felzmann et al. 2019a). The obligations on the design side correspond to the transparency and inspection rights that other stakeholders have, especially users and third parties. The intended target audience of the transparency principles is primarily engineers.

#### Design of AI systems (Phase 1)

When designing AI systems engineers and developers must keep in mind the underpinning values and ethical issues raised by such systems. Mittelstadt and colleagues (2016) provide a systematic overview of the concerns A/IS raises which can be used as a roadmap when designing new systems. The map can be divided into three parts: (1) epistemic concerns relating to the quality of evidence upon which a system relies, (2) normative concerns such as unfair outcomes, and (3) the traceability of a decision. Epistemic concerns should motivate engineers to carefully consider what data is used to reach a certain conclusion and whether it is possible to assess the manner in which a particular conclusion was reached. Normative concerns should motivate engineers to monitor the ethical impact of their systems (Fule and Roddick 2004) and search for errors and biases (Schermer 2011). Lastly, concerns due to challenges regarding traceability (in particular for machine learning algorithms) should prompt engineers to consider how these challenges impact the possibility of transparency and how problematic a lack of traceability might be, including potentially the decision that in some contexts such a lack of traceability might not be justifiable (Mittelstadt et al. 2016). In light of these general considerations regarding transparency in the design process, we propose the following principles for the design phase:

##### Proactivity: Be proactive, Not Reactive (Principle 1)

Technical and organizational measures should be geared towards the realization of transparency from the beginning of the development of the AI system, and not be addressed only after the system design has been completed (Cavoukian 2009). This principle is based on the assumption that technologies by necessity embody values (Nissenbaum 2001) and that only proactive attention to values during the design process can ensure that their realization is not accidentally made more difficult by design decisions. Approaches such as Friedman et al.’s (2008) Value-Sensitive Design, and subject-specific variations, such as Aimee van Wynsberghe’s Care-Centred Value Sensitive Design for Healthcare Robotics (Van Wynsberghe 2013), have conceptualized approaches to design that aim to realize this insight into practice. Systems designers and organizational stakeholders need to deliberate from the outset on how to make information about decision-making standards accessible and comprehensible to those stakeholders with an interest in the system’s decisions, including different categories of users as well as, for instance, regulators. Comprehensibility is tied to the audience-driven communication of information and will be discussed as part of principle 3.

##### Integration: Think of transparency as an integrative process (Principle 2)

The complexity of transparency needs should be taken into account when designing AI systems. It is essential to go beyond a static informational understanding of transparency. Transparency measures should do justice to the complexity of the decision-making process, showing how different aspects of the system contribute to meeting the standards that govern decision-making in the system. As AI systems typically infer from data points and reach conclusions that may go beyond the information contained in this data, companies have to be able to explicate the standards of what they consider acceptable inferences (Wachter and Mittelstadt 2019). Depending on the informational purpose and audience (see principle 3 below), decision-making standards may be presented in the shape of general, high-level principles or more specific and detailed standards.

Taking an integrative view includes awareness of the potential practical impact of drawing on inadequate or insufficiently realized decision-making standards. For instance, IBM (2018) and ICDPPD (2018) acknowledged that while biases and discrimination can never be fully eliminated in AI systems, but that efforts need to be made to reduce or mitigate them. To this end, the decisions made by the AI systems, the data sets and the processes that yield the AI system’s decision, including those of data gathering, data labeling and the algorithms used, should be documented to the best possible standard. This documentation process enables identification of the reasons why an ADM was erroneous, which, in turn, could help anticipate undesired consequences for certain stakeholders, and prevent future mistakes.

##### Audience Focus: Communicate in an Audience-Sensitive Manner (Principle 3)

The developers of AI systems need to do justice to the relational nature of transparency communications. Thus, the responsibility is not just to provide information, but also to consider the likely audience that will receive and interpret the information. As Zarsky (2013) points out, the transparency requirement will vary depending on who the likely information recipient is. While individuals whose personal information is being processed are a primary recipient, it is important to be aware that other stakeholders also need to be considered, such as various system user categories, regulators, watchdogs or the general public, depending on the nature and function of the system under consideration. Information targeted at the general public, where questions about general functioning will be predominant, will likely need to differ from the information provided to the affected individual, where specific information needs regarding decision-making on the individual will be the primary concern. In contrast, the information provided to government agencies, regulators and independent watchdogs, would not only be targeted to their specific requirements but could be potentially more technical in nature, as it can be assumed that such institutions have relevant specialist expertise at their disposal, unlike the general public. “Transparency disclosures may prove more impactful if tailored towards trained third parties or regulators representing public interest as opposed to data subjects themselves” (Mittelstadt et al. 2016, p. 7, with reference to Tutt 2017 and Zarsky 2013). The need to make decisions accessible to affected individuals, the general public, regulatory bodies or watchdogs is also closely linked to preventing public distrust and suspicion; the perceived centrality of trustworthiness is evident for example in the European Commission’s report on Trustworthy AI (HLEG AI 2019).

#### Information on Data Processing and Analysis (Phase 2)

The main focus in this phase is on the information that should be provided to stakeholders with respect to *what* data is processed, *how* it is processed, and what *risks* are associated with this processing. Its core concern is to define what information needs to be disclosed to meet transparency obligations towards stakeholders. The goal of transparent information provision is to achieve explainability of the system and its risks to stakeholders.

According to FAT-ML’s Principles for Accountable Algorithms, explainability means “[e]nsur[ing] that algorithmic decisions, as well as any data driving those decisions, can be explained to end-users and other stakeholders in non-technical terms.” This includes information that allows an explanation of the general functioning of the system, the specific use of data within the system, and individual decisions taken by the system. Explainability as the attempt to open up the black box comprises a number of different albeit closely related goals, directed at potentially different stakeholders (Adadi and Berrada 2018): Justification of decision-making, risk control, system improvement, and discovering new knowledge implicit in the system.

The European Commission’s HLEG AI (2019) acknowledges that there are technical limits to explainability and that sometimes it is impossible to give an explanation of how a system reached a particular decision. The degree of how much explicability thus has to depend on the context, the severity of the consequences of its decision (Adadi and Berrada 2018) and the relevant stakeholders.

##### Data processing: Explain What Data is Being Used and How it is Being Processed (Principle 4)

In light of the potential technical limitations on the explainability of decision-making of complex AI systems, this principle requires to provide an understandable descriptive outline of what data is being used by the system and the ways in which it is being used, including information on what stages of data processing are inspectable and where human discretion, intervention or oversight takes place in the system.

Data can be used in the system for various functions. Zarsky (2013, p. 1532) outlines data use for three different “segments of information flow”: (1) collection of data and aggregation of datasets, (2) data analysis, and (3) usage stage. While the primary transparency interests of stakeholders relate to the usage stage, the functioning of the system and its potential biases are determined by the earlier stages. Accordingly, it can be argued that to “fully meet the transparency requirements at this juncture, access should be provided to the working protocol analysts use for these early segments of the prediction tasks” (Zarsky 2013, p. 1524), including the presentation of (training) data used in the process of analysis.

Input and output transparency can be distinguished (Sunstein 2018). The costs of input transparency tend to be high and the benefits low, not least because of potentially massive volume (Sunstein 2018). During the analysis of data, transparency from a technical point of view could mean that the software used for automated decisions should be disclosed (Zarsky 2013). If custom-made software is used, this becomes more challenging, as such a transparency requirement could jeopardize intellectual property rights. To address these challenges, Kroll and colleagues (2016) discuss systems with properties that can be checked by relevant parties (i.e., regulators or the public) without having to reveal the input data and code. This could help design systems that are transparent about the features that matter for a particular automated decision, while protecting private data and withholding trade secrets.

Transparency could also call for the “disclosure of the actual strategies and practices for using the data” (Zarsky 2013, p. 1526). This should include what the FAT-ML principles define as accuracy, i.e., to “[i]dentify, log, and articulate sources of error and uncertainty throughout the algorithm and its data sources so that expected and worst-case implications can be understood and inform mitigation procedures.” The requirements of presenting such information look differently depending on whether transparency is targeted at experts or lay individuals whose information needs are of a less technical and more general nature. Generally, explainability is understood as making information about a system understandable for the general public.

Transparency information also needs to be provided where human discretion, intervention or oversight occurs. The impact of human decision-making, potentially associated with biases, should be made transparent, for example regarding human input in the creation and selection of training data. The GDPR specifically includes a right not to be subject to automated decision-making that would have a legal or otherwise significant impact on them. Transparency requirements, therefore, need to include statements on where and how such human intervention takes place.

##### Decision-Making standards: Explain decision-making criteria and their justifiability (Principle 5)

In addition to transparency with respect to the descriptive aspects of data processing, attention should be paid to the decision-making criteria (Wachter and Mittelstadt 2019). Such criteria are normative, encoding implicit values. Their explanation and justification can be understood as the first step toward accountability. Transparency of implicit decision-making criteria includes clearly explicating normative implications.

In their analysis of different models for explanations in automated decision-making systems, Wachter and Mittelstadt (2019) explore the difficulties of identifying accurate explanations for black-box decision-making. They outline the respective weaknesses of different approaches to explanation, settling on an understanding of explanation that, rather than aiming for a (necessarily incomplete) accurate descriptive representation, is closely linked to the notion of interpersonal justifiability. Wachter and Mittelstadt (2019, p. 581) propose the following important aspects of justifiability: “(1) why certain data are a normatively acceptable basis to draw inferences; (2) why these inferences are normatively acceptable and relevant for the chosen processing purpose or type of automated decision; and (3) whether the data and methods used to draw the inferences are accurate and statistically reliable”.

Assessment of data processing needs to work both forward, by considering what data sources are linked to the intended outcomes, and backward from results, by considering “the underpinning inferences that determine how we, as data subjects, are being viewed and evaluated by third parties” (Wachter and Mittelstadt 2019, p. 611). The link between data sources and inferences needs to be critically reflected on. For example, in making transparent decision-making criteria for a predictive policing algorithm, it is essential to reflect on the link between the data that is fed into the system, such as the number of drug-related arrests in an area, and the predictive inferences, such as priority level for allocating policing resources to the area.

The following considerations could be relevant to address in the context of justification (Wachter and Mittelstadt 2019, p. 618): the degree of privacy invasiveness of processing, the counter-intuitiveness of the inferences, the specific intentions underpinning the processing, the use of potentially discriminatory features, the potential impact of deriving sensitive data from innocuous data, the normative acceptability of deriving information from source data that was created for different purposes, and the reliability of the inferences.

##### Risk Disclosure: Explain the Risks and risk Mitigation Measures (Principle 6)

This principle focuses on making transparent the risks associated with the operation of the AI system. Risk communication is traditionally an essential element of informed consent (Beauchamp and Childress 2001). Risks associated with automated decision-making have been discussed in the literature (Araujo et al. 2020; Schermer 2011; Bahner et al. 2008). Particularly, automated decision-making poses new risks of “privacy-invasive, discriminatory, and biased decision-making” (Wachter and Mittelstadt 2019, p. 505), of reputation and informational self-determination, and the potential permanence and lack of contestability of problematic records. The potential transfer of inferred data from one organization to another is an additional risk, especially if inferences do not count as personal data. Privacy invasion through algorithmic inferences is a particularly prominent theme in the literature, but issues associated with algorithmic bias and discrimination are also increasingly coming to the fore as significant concerns.

Due to the obscurity of data processing, users have little understanding of risks associated with ADM by the system. Knowledge of risks is important as a precondition to achieving one of the frequently stated goals of transparency, the increase in autonomy and control for users of AI systems. Risks can become apparent to organizations especially when algorithmic prediction shows verifiable failures.

Legally, it is still unclear what remedies individuals will have against problematic ADM. Scholars have highlighted that discriminatory practices and potential chilling effects arising from the employment of such technologies are inadequately covered in the law, like data protection law (Wachter and Mittelstadt 2019; Büchi et al. 2019). Accordingly, it becomes even more important that risks associated with data processing are identified, to allow users a more informed engagement. Transparency on risks requires going beyond the mere impact on privacy (as captured in the GDPR instrument of the Data Privacy Impact Assessment), addressing also risks of bias and discrimination, as well as other risks associated with the failure of ADM. Included in statements on risks should also be information on risk mitigation measures, to allow a better assessment of how significant the residual risks might be. The inclusion of risk-related information arising from any accountability measure would be part of this.

#### Organizational and Stakeholder-Oriented Transparency Management (Phase 3)

Organizational and stakeholder-oriented transparency management is the final phase of the transparency process, referring to requirements of how to engage with societal concerns. The established decision-making standards must be open to reflective scrutiny, must be lived by in practice, and consequences must result if the systems fall short. Organizations can engage with societal expectations in manipulative, adaptive or moral ways (Buhmann et al. 2019); effective transparency management as understood here requires an adaptive response but based on moral foundations.

How exactly responsibility should be assigned for A/IS, is subject to some debate. The mainstream position is represented by ACM (2017), which states that organizations should be held responsible for decisions made by the algorithms that they use, “even if it is not feasible to explain in detail how the algorithms produce their results.” Microsoft (2019), on the contrary, highlights that perhaps AI systems should also be seen to have a kind of “algorithmic accountability.” This appears to suggest a form of non-human responsibility, supported by a continuous focus on the machine as responsible. However, in current societal practice, the locus of accountability still remains with human and organizational actors, rather than artificially intelligent agents.

As outlined above, organizational and stakeholder-oriented transparency management presupposes traceability and auditability of systems. Traceability means that AI systems are designed in a way that allows retracing their decision-making, with a record that allows the reliable reconstruction of relevant processes and decision-making factors and thus the determination of responsibility for certain decisions. Traditionally, responsibility would be linked to the person designing the program. However, such a conception is not suitable for learning algorithms (Mittelstadt et al. 2016; Bozdag 2013). Auditability means that systems need to be designed in a way that allows qualified outsiders to access information on relevant processes and decision-making factors and make judgements on their appropriateness. It also requires that defined processes are in place regarding accountability, and that these are effective in obtaining required responses and achieving change where necessary. The following principles specify our understanding of accountability.

##### Inspectability: Ensure Inspectability and Auditability (Principle 7)

AI systems should enable the inspection of the system’s decision-making through audits. Thus, this principle concerns retrospective, rather than prospective, transparency (Felzmann et al. 2019a; Paal and Pauly 2018; Zerilli et al. 2019).

According to the FAT-ML principles, auditability “enable[s] interested third parties to probe, understand, and review the behavior of the algorithm through disclosure of information that enables monitoring, checking, or criticism, including through provision of detailed documentation, technically suitable APIs (Application Programming Interface), and permissive terms of use.” The FAT-ML description includes specific information targeted towards technically literate stakeholders but would also continue to require that core information about the basis for decisions can be given in descriptions accessible to laypersons.

The principle also includes collaborating with actors and institutions that need to or want to inspect the AI system. In that regard, the designers and creators of the AI system should have in-house capacity not only to walk the auditors through the basic technical logic of the system (e.g., which machine learning approaches and libraries are used) but also be able to work with counterfactuals. Thus, auditors should be able to test the system’s outcomes for a broad variety of typical and atypical scenarios. In addition, inspectability and auditability mean proactive engagement with the scientific community, for example by giving subject-matter experts the necessary access to the infrastructure for testing the systems or by exposing the systems to peer review through journal and conference presentation.

##### Responsiveness: Be Responsive to Stakeholder Queries and Concerns (Principle 8)

This principle demands responsiveness to individual and societal stakeholders beyond the question of the inspectability of individual decisions. Responsiveness means being open to being approached and scrutinized by stakeholders such as journalists, civil society organizations, public administrators, and the general public. It means making it easy for stakeholders to initiate transparency communications if they have legitimate questions, queries, and concerns. It also requires that organizations ensure that transparency queries are responded to meaningfully, with attention to individual cases, and in a timely manner. Representatives of the organization deploying the AI system should approach queries with the default attitude that these queries deserve a serious, timely and individualized response, recognizing the validity of expressed concerns. To facilitate the principle more concretely, contact information should be made easily accessible (informational fairness). For example, instead of merely a short FAQ or chatbot, a clearly visible phone number or email address of a qualified contact person would boost this type of transparency.

Regarding the media, responsiveness means engaging visibly and openly in public debate. For instance, it has been lamented that major technology corporations only speak to journalists “on background,” thus avoiding critical scrutiny (Merchant 2019). Ending this practice would be the first step towards more transparency and could improve the image of these companies among the general public. Reputational concerns are an important mediator for accountability relationships and organizations need to be sensitive to how such concerns emerge in society (Buhmann et al. 2019).

Finally, responsiveness also applies to instances when transparency standards were not met, or when transparency brings to light relevant concerns. Meeting the terms of any sanctions, engaging in critical self-assessment and taking meaningful remedial action are covered by the principle of responsiveness.

##### Reporting: Report Diligently About the System (Principle 9)

This principle specifies that designers of AI systems should make their activities transparent through detailed reporting. The principle calls for the publication of regular reports that give descriptive and aggregate information about the AI system in terms of uptake, use and accuracy, if relevant. For example, developers of criminal justice algorithms and criminal risk assessment tools should publish where the tools are used (i.e., in which jurisdictions), how many decisions the system made (including historical trends), and what the outcomes of the decisions are. Where available, the quality of decision-making should be benchmarked by comparing the results of other accepted decision-making approaches in the field. Such reports could be published on a yearly, quarterly, monthly or daily basis, depending on the importance and timeliness of the information. They could also feature different formats, from a more traditional text document, to a searchable platform giving aggregate information, to an API that allows to extract and scrutinize the raw data. For example, Facebook’s newly established ads library is searchable and frequently updated. It also has an API, which, however, seems to work poorly (Rosenberg 2019). By contrast, Facebook’s community standard enforcement reports, which outline the content moderation performed by the platform (both automatically and manually), and other reports in their transparency section^3^ features a more traditional reporting format, summarizing periods of half a year. The appropriate reporting format should be implemented with the audience’s needs and capabilities in mind. They could be differentiated according to the needs of the most prominent stakeholder groups. Like the other two transparency management principles, this principle demands proactivity regarding both the organization deploying the system and the stakeholders or the general public affected by the system’s operation.

## Discussion

Transparency-by-design is not a methodology in the narrow sense, as it does not offer technical tools that can be directly applied to address transparency. In that sense, TbD cannot provide highly detailed and specific instructions that might be necessary for the technical implementation of the higher-level principles. Instead, the primary purpose of TbD is to offer a framework that can act as a reflection tool for different stakeholders to integrate transparency considerations into their practice. Thus, we propose the TbD framework as a translation and interface between high-level AI ethics principles and guidelines (Jobin et al. 2019) on the one hand and technical implementations for concrete applications on the other hand. For the latter, the TbD framework can guide developers and AI practitioners on-the-ground avoid pitfalls when developing, deploying and evaluating AI-based technologies. Given that the TbD framework is informed by a rich and interdisciplinary assessment of transparency literature and incorporates considerations of both the positive aspects as well as the challenges that come with transparency in ADM systems, we think such a framework to be broadly applicable.

The TbD framework is inspired by privacy-by-design, while also showing important dissimilarities. Privacy is recognized on a constitutional level (at least in Western societies), while transparency is not (at least not to the same extent). However, both are complex and contested concepts, and multiple, and sometimes conflicting, values are attached to both concepts. While most will argue that privacy is necessary and essential, many will argue that the way it is currently protected is not sufficient or not fruitful. Similarly, as shown in our contribution, transparency is both intuitively important in an age where human interaction and societal processes are increasingly mediated by AI systems, but is often hard to grasp and exceedingly difficult to implement, both in light of the intrinsically opaque nature of many AI systems and in light of the necessary complexities of interpretation and variety of affected social interests that relevant social science literature highlights (e.g., Burrell 2016).

Similarly to privacy, transparency is often described as a normative ideal, but at the same time, appears like a fuzzy concept that defies precise linearity. It is tempting to assume that “more transparency is always better”, but this does not seem to be borne out by empirical evidence on the matter, as outlined in Sect. 2.3. When it comes to privacy, Altman (1975) pointed to the fact that an optimal level of privacy is reached when desired and achieved privacy match. There can be not only too little privacy but also too much privacy, in the sense that a person may be isolated and lack social interaction. A similar point can be made about transparency. Too much transparency can be undesirable or even harmful (Ananny and Crawford 2018) and empirical research has shown that too much transparency can have unintended side-effects such as algorithmic disillusionment (Eslami et al. 2018). Thus, we should abstain from naive techno-optimism, or enchanted determinism (Campolo and Crawford 2020), which see (AI) technology as a powerful solution for complex social problems and transparency as something that can be pragmatically integrated into such technologies.^4^ We have shown the practical complexities of transparency in AI systems based on the example of social media content moderation. This example and our discussion of data assemblages and algorithmic culture in the introduction show how transparency has to be seen within broader cultural, social and economic contexts and from a relational perspective.

The optimal level of transparency would be one where desired and achieved transparency match for the respective person or group. Since different stakeholders have different transparency expectations and perceptions, negotiating such an optimum is indeed challenging. While the by-design methodology is quite broad and touches upon certain aspects that go beyond transparency in a narrow sense (e.g., responsibility, fairness, value-sensitivity), integration and application of these wider responsible design principles into the discussion of transparency is essential for achieving practical implementation of the TbD framework and doing justice to the significance of crucial factors that go beyond the informational perspective of transparency. By implementing the TbD framework, organizations and designers may be able to realize the benefits of transparency in a practical way, including fostering trust, facilitating accountability, supporting autonomy, and allowing a higher level of control; while also being conscious of the complexities and contextual embeddedness of transparency with its attendant limitations. This might allow to mitigate some of its inherent risks insofar as it does not neglect the demand-side of transparency, and it is less static and more of an iterative process.

For achieving greater practical applicability in the future and to do justice to the complexities of interacting requirements by the multiple stakeholders identified in the framework, the TbD approach would benefit significantly from the inclusion of these different stakeholders’ perspectives, especially AI developers, engineers, and business practitioners in the field. Developing a more practice based understanding of demands and expectations of transparency by users, developers, and policymakers would test the value of the principles in practice and allow a further refinement of these principles. Still, there are indeed potential reasons why TbD may not be realized; most particularly where organizational incentives misalign with the scope of required transparency, or where there are insufficient economic, social, or normative forces pushing organizations towards its adoption. Such a gap between core organizational incentives and clear, thoughtful, and “win–win” design principles could be an avenue for the TbD framework regulatory adoption; as in the PbD case, this gap has been—on paper—significantly closed by its inclusion in the GDPR.

## Conclusion

Our discussion shows how integrating transparency into the design and implementation process of an AI system is not an easy task. The speed of technological development, the multiple dimensions of the transparency concept, the uncertainty of where transparency is required, how best to approach communication with different stakeholders, and how to embed transparency measures into meaningful and organizationally realistic accountability measures all present challenges to the implementation of this value, despite seemingly general agreement that it is important. Our principles of TbD serve as a proposal on how transparency measures could be concretized. The principles highlight where transparency concerns need to be addressed during the design process by system designers. If the framework proves valuable to this audience, it can be refined into more concrete guidance over time.

Future feedback on the proposal will allow us to reflect on the robustness of the general model and the proposed specific principles and contribute to further fine-tuning the principles. We hope that our proposal can serve as a step towards a more concrete and practically achievable realization of the transparency requirement that is stated in so many guidance documents and now also enshrined in the GDPR.

## Footnotes

1 See https://explainableroboticsystems.wordpress.com/.

2 FAccT (acronym for “**F**airness, **Acc**ountability, **T**ransparency) was formerly called FAT* and refers to the ACM Conference on Fairness, Accountability and Transparency (https://facctconference.org/). This conference has become a leading and interdisciplinary forum for research at the intersection of ethics, machine learning, algorithms and AI. The research community working on these topics has become partly synonymous with the conference.

3 See https://transparency.facebook.com/.

4 Campolo and Crawford (2020) present several compelling reasons to be skeptical of technological transparency approaches. With regards to the field of deep learning, they write: “As researchers work to formulate the field’s definitions and guiding problems, they have come to realize that ‘interpretability’ is not a simple panacea that will inevitably displace discourses like enchanted determinism. Indeed, the most promising research in this area forces us to confront the fact that interpretability is not simply a property of any model or technique but rather only emerges with deep contextual knowledge of the social structures and even histories where they are applied” (p. 14).
